# Factor Xa and thrombin induce epithelial-mesenchymal transition by retinal pigment epithelial cells via PDGF-Rβ signaling

**DOI:** 10.1186/1479-5876-10-S3-P1

**Published:** 2012-11-28

**Authors:** Jeroen Bastiaans, Jan C van Meurs, Conny van Holten-Neelen, Nicole MA Nagtzaam, P Martin van Hagen, Herbert Hooijkaas, Willem A Dik

**Affiliations:** 1 The Rotterdam Eye Hospital, Rotterdam, the Netherlands; 2 Dept. of Immunology, Erasmus MC, Rotterdam, the Netherlands; 3 Dept. of Internal Medicine, Erasmus MC, Rotterdam, the Netherlands; 4 Dept. of Ophthalmology, Erasmus MC, Rotterdam, the Netherlands

## 

Proliferative vitreoretinopathy (PVR) is an inflammatory fibrotic disorder of the retina. Retinal pigment epithelial (RPE) cells contribute to PVR development through uncontrolled proliferation and extracellular matrix production as well as cytokine secretion. Insight into factors that stimulate these processes by RPE in PVR is limited, which may explain the lack of satisfying treatment so far. Blood-retinal barrier breakdown and vascular damage are early pathobiological events of PVR. Vascular damage results in activation of the coagulation system and subretinal fluids from retinal detachment patients have been described to contain high pro-coagulant activity. The effect of coagulation proteases on RPE is however hardly studied so far. Of all proteins involved in the coagulation cascade only factor Xa and thrombin are able to induce various cellular responses via their receptors which are expressed at the cell membranes of various cell types. These receptors, the so-called protease activated receptors (PARs) consist of 4 members: PAR1, -2, -3 and -4. Cleavage of PARs results in the activation of signal transduction pathways that control multiple cellular functions like inflammation, fibrosis and tissue repair.

Here we examine the effect of factor Xa and thrombin on epithelial-mesenchymal transition (EMT) by RPE. EMT is a biological process that allows epithelial cells to undergo multiple biochemical changes that enable them to assume a mesenchymal phenotype. Similar processes have also been described for RPE cells in PVR membranes. Differentiation from an epithelial phenotype into a mesenchymal phenotype results in the loss of epithelial markers like zona occludens (ZO)-1 and the enrichment of mesenchymal markers like α-smooth muscle actin (α-SMA). Another component of PVR development as a result of EMT consists of excessive production of extracellular matrix (ECM) proteins; mainly collagen subtype-I.

In this study we show that factor Xa and thrombin are able to upregulate the expression of pro-fibrotic mediators like PDGF-A, -B, TGF-α and TIMP-1 by RPE cells and induce fibrotic responses known to be involved in the development of PVR. In line with these findings we also show that mainly thrombin but also factor Xa, can induce EMT by RPE via PDGF-B mediated PDGF-Rβ signaling.

